# Inclusion Complexes of Copaiba (*Copaifera multijuga* Hayne) Oleoresin and Cyclodextrins: Physicochemical Characterization and Anti-Inflammatory Activity

**DOI:** 10.3390/ijms18112388

**Published:** 2017-11-18

**Authors:** Jonas Gabriel de Oliveira Pinheiro, Emanuella de Aragão Tavares, Sofia Santos da Silva, Juliana Félix Silva, Yasmim Maria Barbosa Gomes de Carvalho, Magda Rhayanny Assunção Ferreira, Adriano Antunes de Souza Araújo, Euzébio Guimarães Barbosa, Matheus de Freitas Fernandes Pedrosa, Luiz Alberto Lira Soares, Eduardo Pereira de Azevedo, Valdir Florêncio da Veiga Júnior, Ádley Antonini Neves de Lima

**Affiliations:** 1 Department of Pharmacy, Federal University of Rio Grande do Norte, Natal, RN 59012-570, Brazil; jgopinheiro@gmail.com (J.G.d.O.P.); emanuella_ta@hotmail.com (E.d.A.T.); sofiassilvam@hotmail.com (S.S.d.S.); julianafelix_rn@hotmail.com (J.F.S.); euzebiogb@gmail.com (E.G.B.); mpedrosa@ufrnet.br (M.d.F.F.P.); 2 Department of Physiology, Federal University of Sergipe, São Cristóvão, SE 49100-000, Brazil; yasmimgomess@gmail.com (Y.M.B.G.d.C.); adriasa2001@yahoo.com.br (A.A.d.S.A.); 3 Department of Pharmaceutical Sciences, Federal University of Pernambuco, Recife, PE 50740-520, Brazil; magda.ferreira00@gmail.com (M.R.A.F.); lals@gmx.de (L.A.L.S.); 4 Graduate Program in Biotechnology, Laureate International Universities—Universidade Potiguar (UnP), Natal, RN 59056-000, Brazil; azevedoep@hotmail.com; 5 Department of Chemistry, Institute of Exact Sciences, Federal University of Amazonas, Manaus, AM 69077-000, Brazil; valdir.veiga@gmail.com

**Keywords:** *Copaifera multijuga* Hayne, copaiba oil, inclusion complex, cyclodextrin, anti-inflammatory activity

## Abstract

Complexation with cyclodextrins (CDs) is a technique that has been extensively used to increase the aqueous solubility of oils and improve their stability. In addition, this technique has been used to convert oils into solid materials. This work aims to develop inclusion complexes of *Copaifera multijuga* oleoresin (CMO), which presents anti-inflammatory activity, with β-cyclodextrin (β-CD) and hydroxypropyl-β-cyclodextrin (HP-β-CD) by kneading (KND) and slurry (SL) methods. Physicochemical characterization was performed to verify the occurrence of interactions between CMO and the cyclodextrins. Carrageenan-induced hind paw edema in mice was carried out to evaluate the anti-inflammatory activity of CMO alone as well as complexed with CDs. Physicochemical characterization confirmed the formation of inclusion complex of CMO with both β-CD and HP-β-CD by KND and SL methods. Carrageenan-induced paw edema test showed that the anti-inflammatory activity of CMO was maintained after complexation with β-CD and HP-β-CD, where they were able to decrease the levels of nitrite and myeloperoxidase. In conclusion, this study showed that it is possible to produce inclusion complexes of CMO with CDs by KND and SL methods without any change in CMO’s anti-inflammatory activity.

## 1. Introduction

*Copaifera multijuga* Hayne (Leguminosae) is a tropical tree commonly found in Brazil, specifically in the central and western region of Amazon. Among the forms of oleoresins extraction, tapping the trunk of *Copaifera multijuga* Hayne is currently the only method that does not cause the death of the tree. The genus *Copaifera* L. has been studied due to its several biological activities, where the anti-inflammatory effect is the most investigated [1,2,3,4].

Copaiba oil is in an oleoresin that consists of an exudate of acidic resins and volatile compounds [3]. The latter are usually sesquiterpenes, of which β-caryophyllene is the most abundant compound found in the oleoresin [5]. Previous reports have shown that copaiba oil and its main sesquiterpene have some biological activities such as anti-inflammatory [6], antimicrobial [7,8,9], anti-carcinogenic [10], anxiolytic, anti-depressant [11,12] and analgesic activities [13]. Its anti-inflammatory activity is attributed to the synergism of several compounds present in the oleoresin [4,14].

Essential oils or oils with volatile fraction have limitations such as high volatility, low stability, unpleasant taste and smell, among others. To ameliorate these problems, complexation of these essential oils with cyclodextrins (CDs) seems to be a convenient alternative. In addition to solving these limitations, the use of CDs could provide a way of obtaining solid oily materials as a complexation product, which might result in more soluble, stable and better tasting derivatives. Characteristics such as dissolution rate and oral bioavailability depend on the drug’s solubility and, therefore, the increase in the solubility of a poorly soluble compound tends to improve both its dissolution rate and oral bioavailability [15].

CDs are cyclic oligosaccharides with six (α-CD), seven (β-CD), eight (γ-CD), or more (α-1,4-) linked d-glucopyranose units, which have been used as pharmaceutical adjuvants for solubilization of various poorly soluble compounds through the formation of water-soluble complexes. CDs are largely used as pharmaceutical excipient due its non-toxicity, non-absorption in the upper gastrointestinal tract and its ability to be completely metabolized by the colon microflora. However, natural CDs, particularly β-CD, have limited solubility and for this reason it is frequently replaced by hydroxypropyl derivatives of β-CD and γ-CD (i.e., HP-β-CD and HP-γ-CD, respectively) as well as by the randomly methylated-β-CD (RM-β-CD) and sulfobutylether-β-CD (SBE-β-CD) [16,17,18,19,20].

Complexation of oils with CDs is frequently found in the literature. Some examples are the complexation of the essential oil of *Citrus sinensis* (L.) Osbeck with β-CD [21], the leaf essential oil of *Cymbopogon winterianus* Jowitt ex Bor (Poaceae) with β-CD [22], the leaf essential oil of *Lippia grata* with β-CD [23] and the leaf essential oil of *Ocimum basilicum* with β-CD [24].

This work aims to develop inclusion complexes of *Copaifera multijuga* oleoresin (CMO) with β-CD and HP-β-CD, prepared by physical mixture (PM), kneading (KND) and slurry (SL) methods. To investigate the formation of the inclusion complexes, physicochemical characterization was performed through Fourier-transform infrared (FTIR) spectroscopy, scanning electron microscopy (SEM), powder X-ray diffraction (PXRD), thermogravimetric analysis (TGA) and differential scanning calorimetry (DSC). Finally, the anti-inflammatory activity of the inclusion complexes was assessed through carrageenan-induced paw edema in mice, where parameters such as paw thickness, nitrite concentration and myeloperoxidase activity were evaluated.

## 2. Results and Discussion

### 2.1. Quantification of β-Caryophyllene in CMO by Gas Chromatography with Flame Ionization Detector

**Figure 1:**
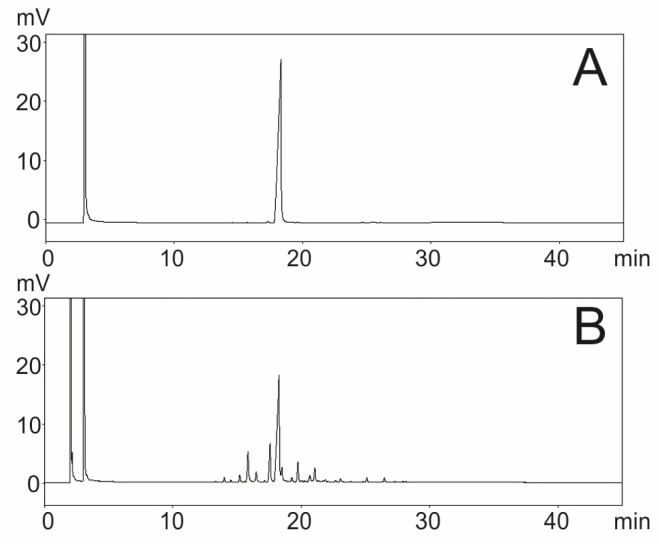
Chromatogram of: β-caryophyllene marker (**A**); and CMO’s volatile fraction (**B**).

The chromatograms of CMO’s volatile fraction and β-caryophyllene marker are shown in Figure 1. The peak assigned to the chemical marker was confirmed by comparing the retention time of the standard (18.24 min) with that of the sample. The data indicate that the sample peak correspond to that attributed to β-caryophyllene. In addition, the results show that β-caryophyllene is the major component of CMO’s volatile fraction. After performing the calculations, it was possible to determine the concentration of β-caryophyllene in the CMO’s volatile fraction to be 45.93 ± 0.54%.

### 2.2. Physicochemical Characterization

#### 2.2.1. Fourier-Transform Infrared (FTIR) Spectroscopy

**Figure 2:**
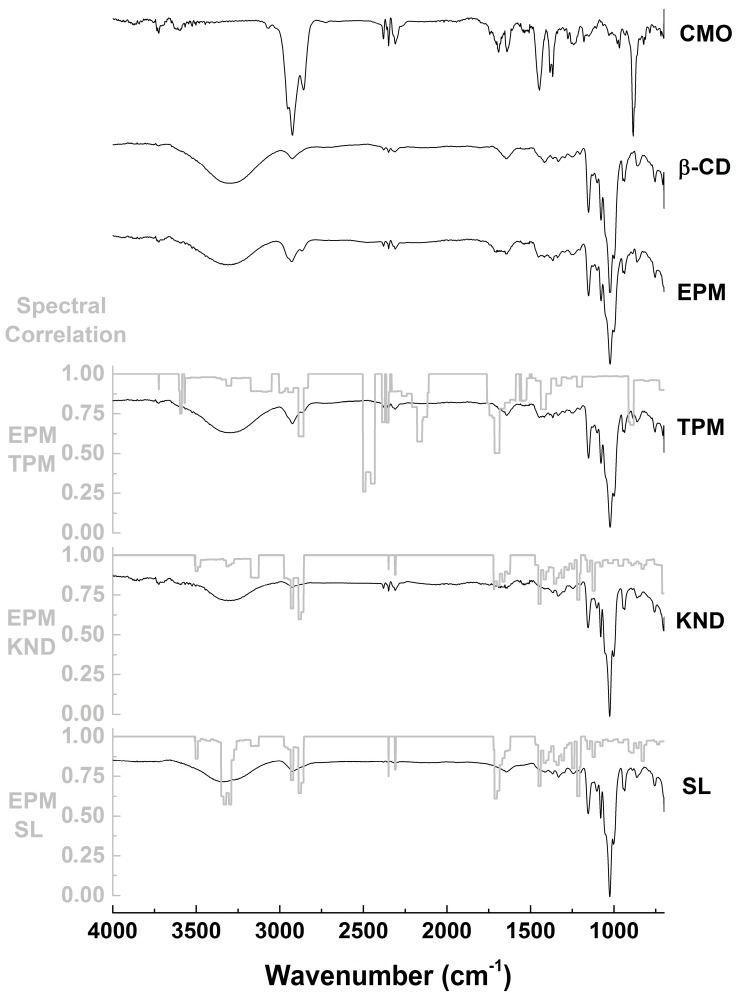
FTIR spectra of CMO and β-CD alone and as inclusion complexes obtained by PM, TPM, KND, and SL as well as their spectral correlations.

**Figure 3:**
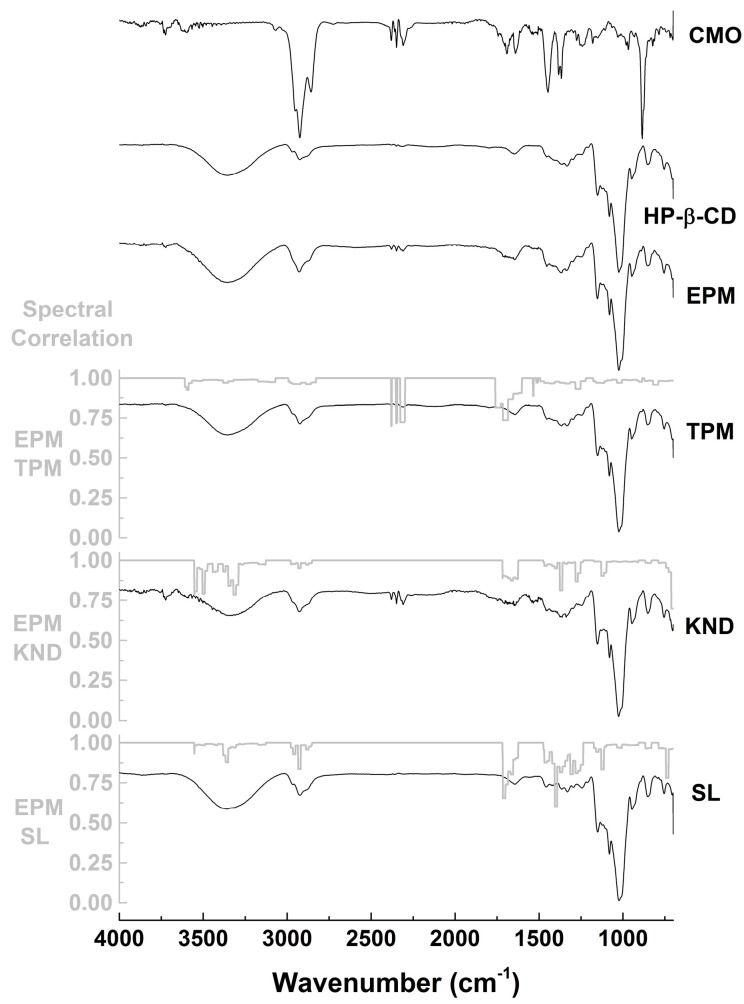
FTIR spectra of CMO and HP-β-CD alone and as inclusion complexes obtained by PM, TPM, KND and SL as well as their spectral correlations.

FTIR spectra and spectral correlation of CMO, β-CD and HP-β-CD alone as well as inclusion complexes prepared by KND and SL are shown in Figure 2 and Figure 3. CMO spectrum shows bands at 2926 and 2856 cm^−1^ (C–H stretching vibrations), 1693 cm^−1^ (C=O stretching vibrations), 1641 cm^−1^ (cycloalkenes C=C stretching vibrations), 1446 cm^−1^ (methyl C–H asymmetric deformation), 1365 cm^−1^ (methyl C–H symmetric deformation) and 887 cm^−1^ (out-of-plane alkenes C–H deformation), which are in accordance with bands reported by Silverstein [25]. β-CD spectrum (Figure 2) shows characteristic bands at 3300 cm^−1^ (O–H stretching vibrations), 2925 cm^−1^ (C–H stretching vibrations), 1151 and 1023 cm^−1^ (C–O–C asymmetric and symmetric stretching vibrations, respectively), as reported by Abarca et al. [26] and Passos et al. [27]. Spectrum of HP-β-CD (Figure 3) shows characteristic bands at 3355 cm^−1^ (O–H stretching vibrations), 2922 cm^−1^ (C–H stretching vibrations), 1151 and 1080 cm^−1^ (C–H and C–O stretching vibrations, respectively), as reported by Medarević et al. [28]. FTIR spectrum of PM of CMO with β-CD (Figure 2) shows characteristic bands attributed to CMO at 2926, 2856, 1693 and 1641 cm^−1^ but at lower intensity when compared to those of the CMO spectrum. On the other hand, the characteristic band at 887 cm^−1^ was suppressed. When comparing to the theoretical physical mixture (TPM) spectrum, PM presents subtle differences due to small changes in the intensities of the correlation bands (Figure 2). According to the spectral correlation, it is possible note that in the KND and SL methods, the characteristic bands of CMO at 2926 cm^−1^ (correlation of 0.67 in KND and 0.73 in SL with PM) and 2856 cm^−1^ (correlation of 0.86 in KND and 0.93 in SL with PM) have a lower intensity in relation to PM and the bands at 1693 cm^−1^ (correlation of 0.81 in KND and 0.66 in SL with PM) and 1641 cm^−1^ (correlation of 0.90 in KND and 0.91 in SL with PM) were totally suppressed (Figure 2).

Similarly, FTIR spectrum of CMO with HP-β-CD prepared by PM (Figure 3) shows characteristic bands assigned to CMO at 2926 and 2856 cm^−1^ but with lower intensity when compared to that of CMO alone and the ones at 1693 and 1641 cm^−1^ appear at very weak intensities. By comparing TPM and PM spectra, slightly differences are observed due to small changes in the correlation bands (Figure 3). When KND and SL are compared to PM, it is possible to observe a discrete decrease in the intensity of the band at 2926 cm^−1^ (correlation of 0.95 in KND and 0.83 in SL with PM) and 2856 cm^−1^ (correlation of 0.98 in KND and SL with PM) in relation to those of PM. In addition, the bands at 1693 cm^−1^ (correlation of 0.90 in KND and 0.74 in SL with PM) and 1641 cm^−1^ (correlation of 0.89 in KND and 0.90 in SL with PM) are enlarged in the KND spectrum and decreased in the SL in relation to PM (Figure 3).

FTIR spectroscopy is a widely used tool to study interactions involving drugs and CDs. By determining the vibrational patterns of CMO and CDs alone, this technique is quite useful in identifying changes in characteristic bands such as disappearance, broadening, variations in peak intensity and/or shifts in their wavenumber, which can be indicative of complexation between CMO and CDs. In addition, spectral correlation is used to facilitate the identification of such changes in the bands as a spectral overlap allows comparing two different spectra, comprising values between 0 and 1. When spectral correlation level falls between 0.9 and 1, there is very strong correlation; between 0.7 and 0.9, there is strong correlation; between 0.5 and 0.7 there is moderated correlation; between 0.3 and 0.5, there is weak correlation; and between 0 and 0.3, the correlation is negligible. The FTIR results indicate that possible interactions took place between CMO and β-CD in the complexes prepared by KND and SL. Changes in the bands attributed to CMO could result from stretching vibrations restriction caused by its inclusion into the CD cavity. In addition, it might be due to weakening of the interatomic bonds caused by the altered environment around these bonds upon complexation [29,30]. On the other hand, the use of HP-β-CD to form inclusion complexes with CMO by KND and SL resulted in spectra with minor differences in relation to those prepared with β-CD, although this finding cannot rule out the possibility of formation of inclusion complexes between CMO and HP-β-CD.

#### 2.2.2. Scanning Electronic Microscopy (SEM)

**Figure 4:**
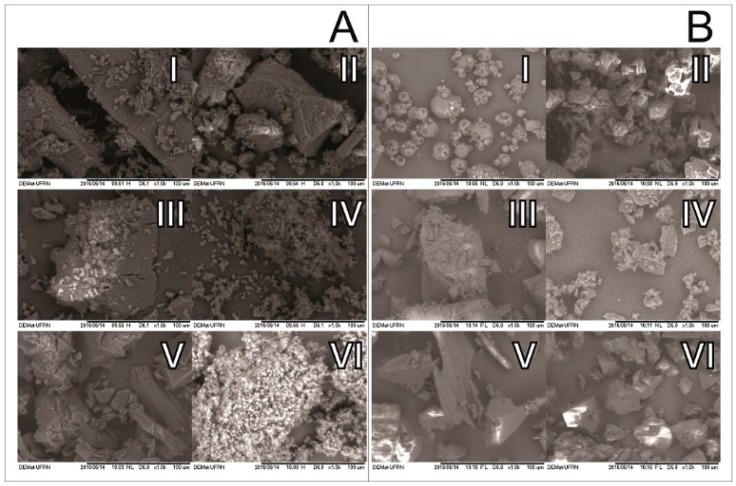
(**A**) SEM micrographs of β-CD (I), PM (II), KND in absence of CMO (III), KND in presence of CMO (IV), SL in absence of CMO (V) and SL in presence of CMO (VI); and (**B**) SEM micrographs of HP-β-CD (I), PM (II), KND in absence of CMO (III), KND in presence of CMO (IV), SL in absence of CMO (V) and SL in presence of CMO (VI). Scale bars: 100 µm.

SEM micrographs of CDs alone and as inclusion complexes with CMO are shown in Figure 4. β-CD surface morphology (Figure 4(AI)) presents particles with crystalline parallelogram structure, as in accordance with Bulani et al. [31]. Micrograph of the PM of CMO with β-CD (Figure 4(AII)) shows particles that are very similar to those of β-CD with crystalline parallelogram structure. Similarly, micrographs β-CD submitted to KND and SL (Figure 4(AIII,AV), respectively) without CMO show the same structure of the β-CD particles, indicating no changes in their morphology after KND and SL processes. However, the surface morphology of the CMO/β-CD complexes obtained by KND and SL (Figure 4(AIV,AVI), respectively) are significantly different from that of β-CD alone. In this case, the appearance of clusters in the particle surfaces was clearly observed. Similar finding was observed by Galvão et al. [21], where the formation of inclusion complexes of *Citrus sinensis* with β-CD drastically changed the surface morphology of β-CD alone.

HP-β-CD morphology appears as spherical amorphous particles (Figure 4(BI)), as previously reported by Melo et al. [32]. SEM micrograph of CMO/HP-β-CD obtained by PM (Figure 4(BII)) clearly indicates that the morphology of the particles has changed. Although the particles still show amorphous characteristics, they are irregularly shaped in the sample obtained by PM. Similarly, by submitting HP-β-CD through the KND and SL processes without CMO, the resulting particles show irregular shapes (Figure 4(BIII,BV), respectively). SEM micrographs of CMO/HP-β-CD particles obtained by KND and SL (Figure 4(AIV,AVI), respectively) show irregular shapes, which are very similar to those obtained by PM and without CMO. Similar findings were also observed by Michalska et al. [33], even though some difference in morphology was observed between the PM and the inclusion complexes. The fact that the morphologies of the particles are quite similar to those obtained by PM and those obtained by both KND and SL does not mean that no interaction occurred between CMO and the CDs and therefore, additional studies need to be performed in order to confirm the formation of inclusion complexes. However, modifications in the shape and aspect of surface morphology have been suggested as an indication of inclusion complex formation with CDs [34,35].

#### 2.2.3. Powder X-Ray Diffraction (PXRD)

**Figure 5:**
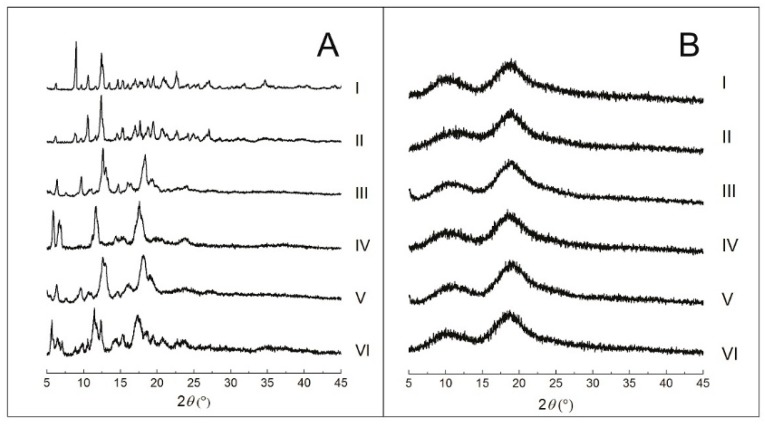
(**A**) Diffractograms of β-CD (I), PM (II), KND in absence of CMO (III), KND in presence of CMO (IV), SL in absence of CMO (V) and SL in presence of CMO (VI); and (**B**) diffractograms of HP-β-CD (I), PM (II), KND in absence of CMO (III), KND in presence of CMO (IV), SL in absence of CMO (V) and SL in presence of CMO (VI).

Crystallographic patterns of each individual component as well as complexed with CDs are presented in Figure 5. β-CD diffractogram (Figure 5(AI)) shows the main crystal reflections at 9.02°, 10.62°, 12.40°, 19.52°, 20.96° and 22.62°. The diffractogram of the PM of CMO/β-CD (Figure 5(AII)) presents the same crystallographic pattern of β-CD alone (Figure 5(AI)), with only subtle differences in the intensity of the peaks at 9.02° and 12.40°. The crystallographic pattern of β-CD submitted to KND (Figure 5(AIII)) and SL (Figure 5(AV)) were different from those of β-CD alone and PM, where both present peaks at 6.45°, 9.67°, 12.60° and 18.28°. On the other hand, the CMO/β-CD complex obtained by KND shows crystallographic pattern (Figure 5(AIV)) different from that obtained by SL (Figure 5(AVI)). In addition, the association of CMO and β-CD by KND and SL show crystallographic profiles different from those of β-CD and PM. These findings corroborate with those of Abarca et al. [26], where they showed that the interaction of cyclodextrin with essential oils caused significant changes in the PXRD profile of the individual cyclodextrin.

HP-β-CD diffractogram (Figure 5(BI)) shows two broad halos, characteristic of an amorphous material. Different from what occurred with β-CD, the samples prepared with HP-β-CD through PM (Figure 5(BII)), KND without and with CMO (Figure 5(BIII,BIV), respectively) and SL with and without CMO (Figure 5(BV,BVI)) show the same amorphous profile of that of HP-β-CD. Even though the results of PXRD did not show any difference between the CMO/HP-β-CD samples and HP-β-CD alone, SEM results indicate that there were changes in morphology.

In fact, changes in the position of diffraction peaks or their disappearance are usually associated with the formation of inclusion complexes. However, to characterize complexation, the inclusion complex pattern must be different from that of PM [36,37].

#### 2.2.4. Thermogravimetric Analysis (TGA)

**Figure 6:**
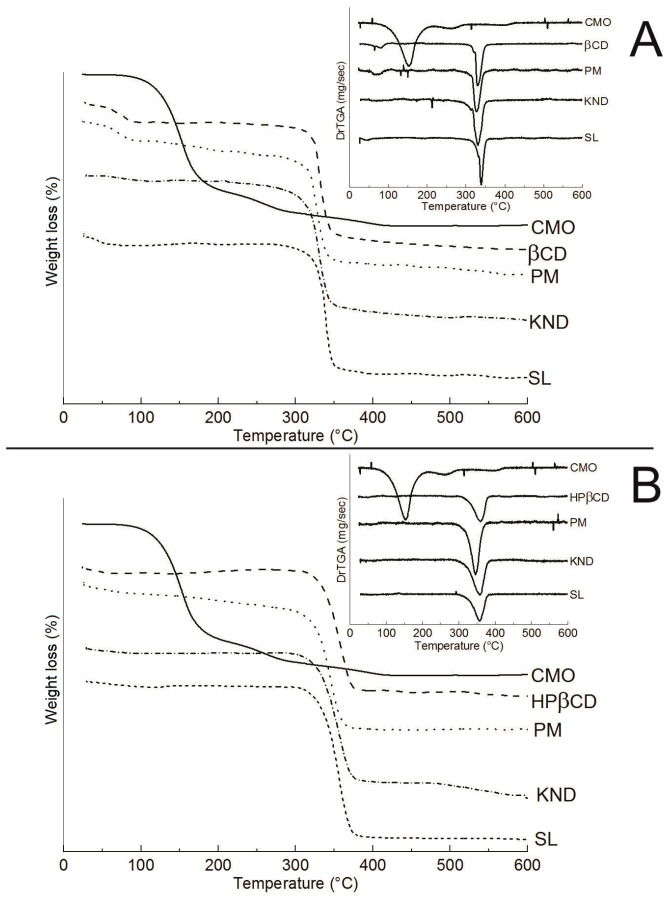
(**A**) TG/DTG curves for CMO, β-CD and CMO/β-CD obtained by PM, KND and SL; and (**B**) TG/DTG curves for CMO, HP-β-CD, and CMO/HP-β-CD obtained by PM, KND and SL.

TG/DTG curves for all samples are shown in Figure 6 and Table 1. CMO presents a mass loss of 74.9% within the temperature range of 25–200 °C (Figure 6A,B) due to its volatilization and another mass loss of 23.2% in the 200–600 °C range. β-CD (Figure 6A) presents two events of mass loss: the first one of 13.6% within the temperature range of 25–200 °C, which is associated with the release of water molecules from its surface [38]; and a second event of 79.4% of mass loss within the temperature range of 200–400 °C, followed by degradation. The curves also showed that the method of complexation influenced on the TG/DTG results, as a mass loss of 18.1% was observed between 25 and 200 °C in the sample obtained by PM, whereas a mass loss of only 4.06% and 6.34% were achieved in the samples obtained by KND and SL, respectively (Figure 6A). The higher extent of mass loss of PM in comparison to the samples obtained by KND and SL seems to indicate that part of CMO was volatilized, which is characteristic of non-interaction with cyclodextrin. In the KND and SL methods, the mass losses are significantly lower within the same temperature range, which suggests a replacement of water molecules inside the β-CD cavity for a hydrophobic compound [39].

The TG/DTG curve for HP-β-CD (Figure 6B) presents a mass loss of 2.93% within the temperature range of 25–200 °C, which is also associated with the release of water molecules from HP-β-CD surface. In addition, HP-β-CD losses 79.79% and 3.88% of its mass in the temperature ranges of 200–400 and 400–600 °C, respectively. However, CMO/HP-β-CD samples obtained by PM, KND and SL yielded mass losses of 11.29%, 3.61% and 2.63%, respectively (Figure 6B). Similar to the results obtained with β-CD, the PM of CMO with HP-β-CD presents higher extent of weight loss than those samples obtained by KND and SL, which seems to indicate that the method of PM leads to no interaction between CMO and HP-β-CD. Moreover, when the temperature range of 400–600 °C is considered, the CMO/HP-β-CD prepared by KND yielded a percentage of mass loss ten times higher (10.62%) than that obtained by SL (1.10%), as showed in Table 1.

#### 2.2.5. Differential Scanning Calorimetry (DSC)

**Figure 7:**
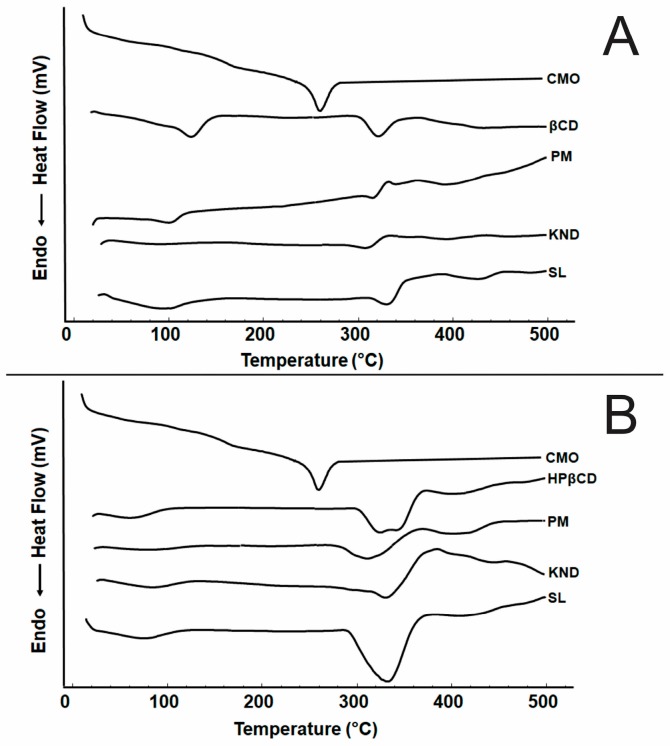
(**A**) DSC curves for CMO, β-CD and CMO/β-CD systems obtained by PM, KND and SL; and (**B**) DSC curves for CMO, HP-β-CD and CMO/HP-β-CD systems obtained by PM, KND and SL.

DSC curves are shown in Figure 7. CMO shows one endothermic peak at 279.39 °C (Figure 7A,B) corresponding to its volatilization. DSC curve for β-CD shows two endothermic peaks. The first one, within the temperature range of 34–155 °C, as observed in the TG/DTG (Figure 6A), is due to the release of water molecules from its surface. The second endothermic peak appears in the temperature range of 303–346 °C, as observed in TG/DTG (Figure 6A), which is associated with β-CD decomposition. Regarding the DSC curves for CMO/β-CD systems obtained by PM and SL, a first event associated with the release of water molecules from β-CD’s cavity is observed (Figure 7A), where it occurs at a lower temperature in the sample obtained by SL. The DSC curve for CMO/β-CD obtained by KND shows the disappearance of the event between 34 and 155 °C, which is also due to the release of water molecules from the β-CD’s cavity, as well as the disappearance of the event related to CMO’s volatilization in the PM, KND and SL methods, indicating interaction between CMO and β-CD. The DSC curve for HP-β-CD shows two endothermic peaks: the first one in the 34–110 °C range, which is associated with the release of water molecules; and a second one at 301–375 °C related to its decomposition. Regarding the CMO/HP-β-CD systems, the thermal event related to CMO’s volatilization disappears, regardless of the method of CMO/HP-β-CD preparation, indicating interaction between CMO and HP-β-CD.

DSC is a powerful analytical tool for identifying interactions between drugs and CDs. As a rule of thumb, changes in DSC curves such as reduction or broadening of peaks as well as shifting to lower temperatures, are a consequence of complexation between the drug and the CD [29,40].

### 2.3. Carrageenan-Induced Hind Paw Edema Test

**Figure 8:**
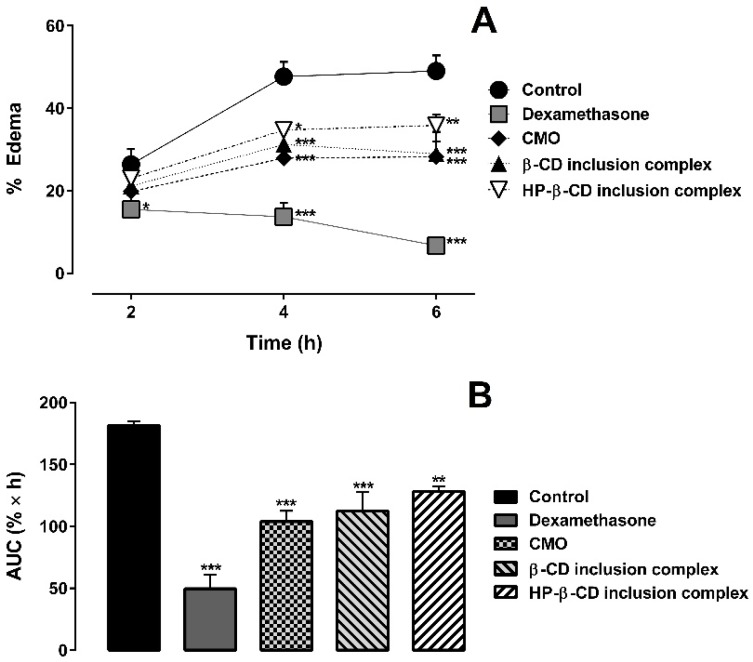
Effect of CMO (alone and complexed with cyclodextrins) on carrageenan-induced paw edema in mice: (**A**) Time-course of the increase in paw thickness along 6 h. * *p* < 0.05, ** *p* <0.01 and *** *p* < 0.001 when compared to control (administration of vehicle alone) in two-way ANOVA followed by Bonferroni’s test. (**B**) Area under the curve for paw edema at 6 h after carrageenan injection. Data are presented as mean ± SEM (*n* = 5/group).

The anti-inflammatory activities of CMO, dexamethasone (2 mg∙kg^−1^), and the inclusion complexes of CMO with β-CD and HP-β-CD (prepared by SL method) were investigated using carrageenan-induced hind paw edema test in mice, as shown in Figure 8. CMO alone induced evident paw edema inhibition, corroborating with previous studies that use crude CMO [1,2,3,4]. In addition, the inclusion complexes of CMO with β-CD and HP-β-CD maintained the paw edema inhibition of CMO (Figure 8), indicating that the complexation with the CDs did not affect CMO’s anti-inflammatory activity. Area under the curve after 6 h of paw edema is shown in the Figure 8B.

Indirect measurement of nitric oxide (NO) production can be made by determining the nitrite levels. NO is a potent vasodilator, which is involved in the inflammation process. NO has the ability to increase vascular permeability and cause edema [41]. Myeloperoxidase (MPO) activity indirectly relates to neutrophil migration and inflammation, which means that, if a given compound decreases MPO activity, it has a potential anti-inflammatory effect [42].

**Figure 9:**
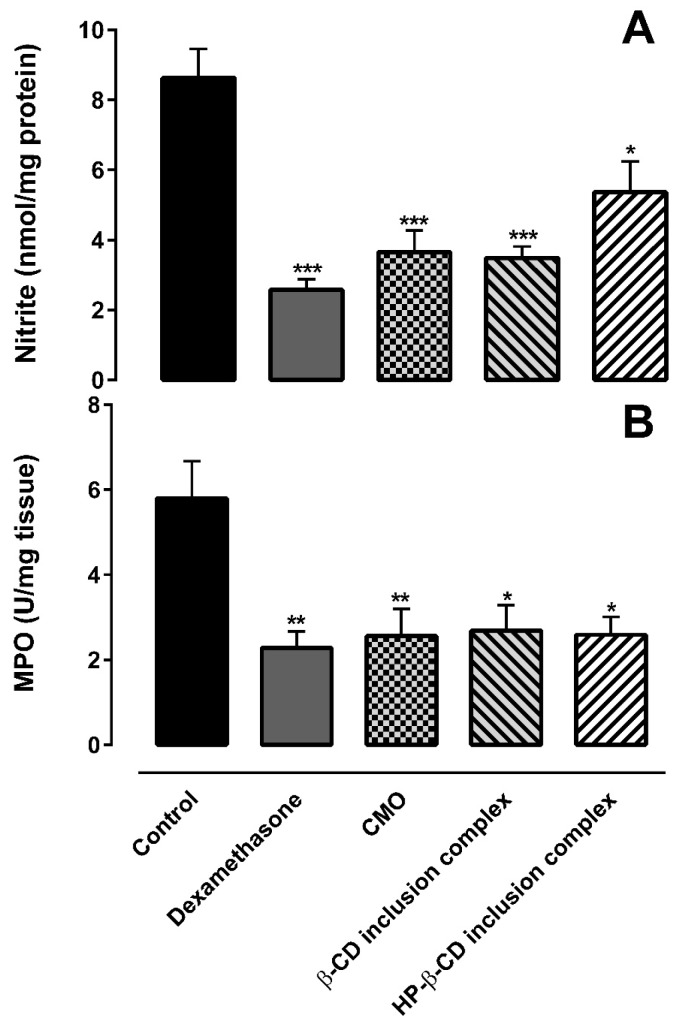
(**A**) Effect of CMO (alone and complexed with cyclodextrins) on the level of nitrite in the hind paws of mice in carrageenan-induced paw edema. * *p* < 0.05 and *** *p* <0.001 when compared to control (administration of vehicle) in one-way ANOVA followed by Tukey’s test. (**B**) Effect of CMO (alone and complexed with cyclodextrins) on MPO activity in the hind paws of mice in carrageenan-induced paw edema. * *p* < 0.05 and ** *p* <0.01 when compared to control (administration of vehicle) in one-way ANOVA followed by Tukey’s test. Data are shown as mean ± SD (*n* = 5/group).

Nitrite concentration is shown in Figure 9A. CMO alone and as inclusion complexes with β-CD and HP-β-CD, as well as dexamethasone, yielded lower concentrations of nitrite in relation to the control group. Similarly, MPO activity of CMO alone and as inclusion complexes with β-CD and HP-β-CD, as well as dexamethasone, presented lower MPO activity than that of the control group (Figure 9B).

Based on the results of the carrageenan-induced hind paw edema test, we can assume that the complexation of CMO with the CDs through SL method did not interfere in the anti-inflammatory activity of CMO.

## 3. Materials and Methods

### 3.1. Materials

*Copaifera multijuga* oleoresin (CMO) was collected from the Reserva Florestal Ducke, at the Instituto Nacional de Pesquisas da Amazônia (INPA) during the summer of 2006, where this is the only species of *Copaifera* available. Exsiccate was properly deposited at the INPA herbarium. β-CD, HP-β-CD, albumin, Coomassie brilliant blue G-250, hexadecyltrimethylammonium bromide, naphthyl ethylenediamine dihydrochloride, o-dianisidine, sodium nitrite, sulfanilamide, Tween 80 and λ-carrageenan were purchased from Sigma-Aldrich^®^ (St. Louis, MO, USA). β-caryophyllene [*trans*-(−)-caryophyllene] standard was purchased from ChromaDex^®^ (Irvine, CA, USA). Hydrogen peroxide was purchased from Merck (Darmstadt, Germany). All experiments were carried out using purified water (<1.3 μS) obtained by reverse osmosis. All reagents were of analytical grade. Phosphate buffered saline (PBS) was used in this study and was prepared with the following constituents: 137 mM NaCl, 3 mM KCl, 1.5 mM KH_2_PO_4_ and 10 mM Na_2_HPO_4_, pH 7.4.

### 3.2. Quantification of β-Caryophyllene on CMO by Gas Chromatography with Flame Ionization Detector (GC-FID)

First, 1.5 g of CMO was subjected to distillation under 90 °C for 90 min, where the volatile fraction was collected in an Eppendorf^®^ tube containing anhydrous sodium sulfate followed by dilution with 2 mL of dichloromethane. The β-caryophyllene quantification was performed by injection of β-caryophyllene [t*rans*-(−)-caryophyllene] (standard) with subsequent comparison of the retention times. Exactly 3.0 μL of the sample was injected into a Supelco^®^ DB-5 column (30 cm, 25 mm × 25 μm) with 1:50 split ratio and helium as carrier gas (1.7 mL∙min^−1^). The temperature was set to 120 °C for 5 min at the initial isotherm followed by an increase of 2 °C∙min^−1^ until 160 °C and 20 °C∙min^−1^ until 240 °C, where it was held for 5 min. The injector and detector temperatures were 250 °C and 260 °C, respectively.

### 3.3. Preparation of Inclusion Complexes

Inclusion complexes of CMO with either β-CD or HP-β-CD were prepared by tree different methods: physical mixture (PM), kneading (KND) and slurry (SL), following the molar ratio of 1:1 (CMO:CD), considering that the molecular weight of CMO is equal to that of β-caryophyllene (204.35 g∙mol^−1^) and molecular weights of β-CD and HP-β-CD are 1134.98 and 1396 g∙mol^−1^, respectively.

#### 3.3.1. Physical Mixture (PM)

CMO and either β-CD or HP-β-CD was separately weighed following the molar ratio of 1:1 and submitted to homogenization using mortar and pistil. The obtained sample was stored in desiccator until analysis.

#### 3.3.2. Kneading (KND)

KND method was performed according to the procedure described by Galvão et al. [21] with minor modifications. Briefly, CMO and either β-CD or HP-β-CD were submitted to homogenization using mortar and pistil followed by the addition of purified water until the formation of a paste (approximately 10 mL of water for each 1 g of the CMO/CD mixture). The obtained sample was dried at 100 °C until constant weight. The dried sample was stored in desiccator until analysis.

#### 3.3.3. Slurry (SL)

SL method was performed following the procedure described by Quintans-Júnior et al. [43] with minor modifications. Briefly, CMO and either β-CD or HP-β-CD were properly weighted and transferred to a recipient to which purified water was added at a ratio of 3:4 (*v*:*w*) relative to the weight of CMO/CD, followed by stirring for 36 h at ambient temperature. The obtained sample was dried at 100 °C until constant weight, followed by storage in desiccator until analysis.

### 3.4. Physicochemical Characterization

To confirm the formation of the inclusion complexes, physicochemical characterization were performed through FTIR spectroscopy, SEM, PXRD, TGA and DSC analysis.

#### 3.4.1. Fourier-Transform Infrared (FTIR) Spectroscopy

FTIR spectroscopic analysis was performed in IR Prestige-21 instrument, in which the samples were placed directly into the Attenuated Total Reflectance (ATR) accessory. FTIR spectra were obtained between 4000 and 700 cm^−1^ using 16 scans and spectral resolution of 4 cm^−1^. To compare different spectra, a spectral correlation study was performed with an ad hoc algorithm through spectral overlap, as described by Lavor et al. [44]. Based on the individual spectra of CMO and CDs, a combined spectrum was created consisting on the sum of the CMO and CD spectra, resulting in the theoretical physical mixture (TPM) spectrum, in which there is no interaction between CMO and each CD. Next, TPM spectrum was compared with that of PM. The same correlation was performed between PM spectrum and those of KND and SL.

#### 3.4.2. Scanning Electron Microscopy (SEM)

The samples were mounted on SEM specimen stubs with double adhesive tape, where the micrographs were obtained in a Hitachi TM-3000 Tabletop Microscope, operated at an accelerating potential of 15 kV, under reduced pressure with magnification of 1000×.

#### 3.4.3. Powder X-ray Diffraction (PXRD)

Diffractograms were obtained using a Bruker D2 Phaser device with CuKα radiation (λ = 1.54 Å) and a Ni filter, generated at 30 kV and 10 mA, with step 0.02° using a Lynxeye detector.

#### 3.4.4. Thermogravimetric Analysis (TGA)

Thermograms were obtained using a thermobalance, model TGA-50 (Shimadzu^®^, Kyoto, Japan), in the temperature range of 30–600 °C, using alumina crucibles with approximately 2 mg of sample under dynamic nitrogen atmosphere (50 mL∙min^−1^) and heating rate of 10 °C∙min^−1^. TG/DTG was conferred using CaC_2_O_4_∙H_2_O standard in conformity to ASTM.

#### 3.4.5. Differential Scanning Calorimetry (DSC)

DSC analysis was performed in a DSC-50 (Shimadzu^®^) using approximately 2 mg of the sample in aluminum crucibles under dynamic nitrogen atmosphere (50 mL∙min^−1^) and heating rate of 10 °C∙min^−1^ within the temperature range of 30–500 °C. The calibration of DSC was carried out with indium (m.p. 156.6 °C; ΔH_fus._ = 28.54 J∙g^−1^) and zinc (m.p. 419.6 °C).

### 3.5. Evaluation of Anti-Inflammatory Activity

#### 3.5.1. Animals

Male and female Swiss mice (30–35 g, 6–8 weeks-old) were kept under standard environmental conditions, with access to water and food ad libitum. Experimental protocols were followed in agreement with the recommendations of the National Council for the Control of Animal Experimentation of Brazil (CONCEA) and the International Guiding Principles for Biomedical Research Involving Animals of the Council of International Organizations of Medical Sciences (CIOMS). In addition, the experimental protocols were approved by the Ethics Committee on Animal Use from the Universidade Federal do Rio Grande do Norte (protocol No. 010/2015). The animals were food-fasted (only water ad libitum) 18 h prior to experiment. At the end of the experiment, the animals were euthanized by intraperitoneal injection of sodium thiopental 100 mg∙kg^−1^ associated with lidocaine 10 mg∙kg^−1^.

#### 3.5.2. Induction of Paw Edema

The influence of CMO complexation with cyclodextrins in its anti-inflammatory activity was evaluated on hind paw edema induced by λ-carrageenan, as previously described in the literature with some few modifications [45]. Groups of animals (*n* = 5) were treated orally (p.o.) with Tween 80 at 3% in PBS (10 mL∙kg^−1^, vehicle control), dexamethasone (2 mg∙kg^−1^, standard anti-inflammatory agent), CMO alone (100 mg∙kg^−1^) as well as inclusion complexes with β-CD and HP-β-CD (at doses equivalent to 100 mg∙kg^−1^). The dose of CMO was chosen based on dose-effect studies (results not shown) and in previous reports with oleoresins [46]. Sixty minutes after treatment, the animals received a subplantar injection of 50 μL of 1% λ-carrageenan (500 μg/paw) at the right hind paw. Increase in paw thickness was measured with a digital caliper (Digimess, São Paulo, Brazil) at 2, 4 and 6 h post-carrageenan injection. Edema was expressed as the percentage of the difference between the paw thickness after (at respective time points) and before (basal values) carrageenan injection.

At the end of the experiment, the animals were euthanized and their right hind paws were collected. Paw skin tissues were weighed, chopped and homogenized in PBS (1 mL of buffer for each 50 mg of tissue), then sonicated in an ice bath for 30 s. The samples were centrifuged at 10,000× *g* at 4 °C for 10 min, where the supernatants were used for quantification of nitrite, as an indirect measure of nitric oxide (NO) production (see Section 3.5.3). The pellets were further used for myeloperoxidase (MPO) extraction and quantification, as an indirect measurement of neutrophil migration (see Section 3.5.4). Protein content was determined by Bradford protein assay using albumin as standard [47].

#### 3.5.3. Nitrite Determination

Nitrite was quantified in mice’s paws by Griess reaction, as previously described in literature with some few modifications [48]. Briefly, 50 μL of each paw supernatant was mixed with 50 μL of 1% sulfanilamide in 5% phosphoric acid and incubated in the dark at 22 °C for 5 min. Then, 50 μL of 0.1% naphthyl ethylenediamine dihydrochloride was added and the absorbance was measured at 540 nm on a microplate reader (Epoch-Biotek, Winooski, VT, USA). The amount of nitrite was calculated using a sodium nitrite standard curve and expressed as nmol of nitrite per mg of protein.

#### 3.5.4. Myeloperoxidase (MPO) Activity

Pellets obtained from the centrifugation process (see Section 3.5.2) were used for extraction of MPO enzyme, as previously reported [49,50]. First, the pellets were homogenized in 0.5% hexadecyltrimethylammonium bromide in 50 mM potassium phosphate pH 6.0 (1 mL of solution for each 50 mg of tissue). Then, the samples were sonicated in ice bath for 30 s and submitted to three freeze-thaw cycles followed by centrifugation at 10,000× *g* for 10 min at 4 °C, where 20 μL was pipetted and mixed with 200 μL of 50 mM potassium phosphate pH 6.0 containing 0.0005% hydrogen peroxide and 0.167 mg∙mL^−1^ o-dianisidine. Finally, the MPO activity was colorimetrically determined using a microplate reader (Epoch-Biotek, Winooski, VT, USA) through kinetic reading at 1 min intervals, during 5 min, at 460 nm. One unit of MPO activity was equivalent to the consumption of 1 μmol of hydrogen peroxide per minute, considering that 1 μmol of hydrogen peroxide gives a change in absorbance of 1.13 × 10^−2^ per minute [45]. The results were expressed as units of MPO activity per milligram of paw tissue.

#### 3.5.5. Statistical Analysis

All results are presented as mean ± standard error of mean (SEM), with 5 animals per each group. One-way ANOVA followed by Tukey’s test or Two-way ANOVA followed by Bonferroni’s test were performed using GraphPad Prism version 5.00 (San Diego, CA, USA). *p* Values less than 0.05 were considered significant.

## 4. Conclusions

Based on the results of FTIR, SEM, PXRD, TGA and DSC analysis, CMO successfully complexed with both β-CD and HP-β-CD by KND and SL methods, where β-CD seems to be more efficient in forming inclusion complexes with CMO. Carrageenan-induced hind paw edema test showed that the inclusion complexes of CMO with β-CD and HP-β-CD prepared for SL maintained the anti-inflammatory activity of the former, as evidenced by the reduction of different inflammatory parameters, such as paw edema, nitric oxide production and neutrophil recruitment in mice paws.

It is of great importance to conduct future studies aiming at developing pharmaceutical dosage forms containing these inclusion complexes, where the compatibility of the CMO/CD system with pharmaceutical excipients will certainly be investigated.

## 5. Patents

Inclusion complexes of copaiba oleoresin (Copaifera genus) with cyclodextrins and their application in the treatment of inflammatory diseases. 2016, Brazil. Patent: Privilege of Innovation. Record number: BR1020160251087, Record institution: INPI-National Institute of Industrial Property, Deposit: 10/26/2016.

## Figures and Tables

**Table 1:** Mass losses (%) under the temperature ranges of 25–200 °C, 200–400 °C and 400–600 °C.

Sample	Δm_1_ (%)	Δm_2_ (%)	Δm_3_ (%)
	25–200 °C	200–400 °C	400–600 °C
CMO	74.99	22.62	0.55
β-CD	13.60	79.42	6.02
PM (β-CD)	18.09	70.74	6.68
KND (β-CD)	4.06	86.40	5.34
SL (β-CD)	6.34	75.79	1.90
HP-β-CD	2.93	79.79	3.88
PM (HP-β-CD)	11.29	80.06	0.00
KND (HP-β-CD)	3.61	84.09	10.62
SL (HP-β-CD)	2.63	82.96	1.10

## Abbreviations

β-CD	β-Cyclodextrin
CDs	Cyclodextrins
CMO	*Copaifera multijuga* oleoresin
DSC	Differential scanning calorimetry
FTIR	Fourier-transform infrared
HP-β-CD	Hydroxypropyl-β-cyclodextrin
HP-γ-CD	Hydroxypropyl-γ-cyclodextrin
KND	Kneading
PM	Physical mixture
PXRD	Powder X-ray diffraction
RM-β-CD	Randomly methylated-β-cyclodextrin
SBE-β-CD	Sulfobutylether-β-cyclodextrin
SEM	Scanning electronic microscopy
SL	Slurry
TGA	Thermogravimetric analysis
TPM	Theoretical physical mixture
γ-CD	γ-Cyclodextrin
